# Identifying nutraceutical targets to treat polycystic ovary syndrome using graph representation learning

**DOI:** 10.1038/s44294-025-00117-4

**Published:** 2025-12-01

**Authors:** Simon Hanassab, Joshua Southern, Ayomide V. Olabode, Ivan Laponogov, Michael Bronstein, Alexander N. Comninos, Thomas Heinis, Ali Abbara, Chioma Izzi-Engbeaya, Kirill Veselkov, Waljit S. Dhillo

**Affiliations:** 1 Department of Computing, Imperial College London, London, UK; 2 Section of Endocrinology and Investigative Medicine, Department of Metabolism, Digestion, and Reproduction, Imperial College London, London, UK; 3 UKRI Centre for Doctoral Training in AI for Healthcare, Imperial College London, London, UK; 4 Department of Surgery & Cancer, Imperial College London, London, UK; 5 Department of Computer Science, University of Oxford, Oxford, UK; 6 AITHYRA, Austrian Academy of Sciences, Vienna, Austria; 7 Department of Endocrinology, Imperial College Healthcare NHS Trust, London, UK

**Keywords:** Reproductive disorders, Reproductive biology, Endocrine reproductive disorders

## Abstract

Polycystic ovary syndrome (PCOS) is a complex, multifactorial, and polygenic disorder. Here, we employed machine learning (ML) techniques to analyze large open-source datasets to identify bioactive molecules in foods and pharmacological agents that interact with genes and biological functions central to PCOS pathophysiology. We selected 13 PCOS-associated genes as targets, and the network propagation algorithm systematically identified bioactive molecules that interact with pathways relevant to PCOS. Among the top-ranked molecules, epicatechin-3-gallate (found in green tea) and 24-methylenecycloartan-3-ol (found in almonds) were newly identified, with green tea and almonds previously demonstrated to have anti-androgenic and anti-inflammatory properties. Validation of the ML pipeline with clinically available drugs revealed significant interactions with gonadotropin-releasing hormone receptor modulators, consistent with their established role in PCOS pathophysiology. These findings identify novel therapeutic targets for further research in precision nutrition and drug repurposing for PCOS treatment.

## Introduction

Polycystic ovary syndrome (PCOS) is the most prevalent endocrinopathy, impacting 11–13% of pre-menopausal women worldwide^1^. Due to its complex pathophysiology and varied presentation, diagnosis and treatment are significant challenges, resulting in suboptimal health outcomes in people with this condition. PCOS is often characterized by a dysfunctional hypothalamic-pituitary-ovarian axis, insulin resistance, and hyperandrogenism^2^. According to the most recent international guidelines^1^, patients must fulfill at least two of the following criteria: clinical and/or biochemical hyperandrogenism; ovulatory dysfunction and/or oligo-amenorrhea; and, polycystic ovaries on ultrasound or, in adults only, elevated anti-Müllerian hormone (AMH)^1^.

PCOS is a complex polygenic disorder with several factors contributing to its pathogenesis^2,3^. Twin studies have demonstrated that heritability in PCOS exceeds 70%^4^. Several genome-wide association studies (GWAS) have identified loci that are associated with PCOS in different populations^2^, wherein the AMH gene, insulin receptor (INSR) gene, and lamin A/C (LMNA) gene are associated with the development of polycystic ovaries^5,6^. Mendelian randomization (MR) studies have suggested additional causal factors of PCOS, including increased body mass index (BMI), insulin resistance, and fasting insulin, and reduced sex hormone-binding globulin (SHBG)^2^. The phenotypic presentations of PCOS are influenced by epigenetic and genetic factors^2^, whereby the activity of associated genes in different environments can lead to variations in patient symptoms and signs. In clinical practice, phenotypic variation can lead to substantial delays in PCOS diagnosis and treatment^2,7^. Furthermore, due to the variation in presentation and symptoms experienced by patients, it remains difficult to identify a universally effective treatment^8^.

PCOS guidelines recommend lifestyle interventions as first-line treatment; a 5% weight reduction has been shown to alleviate metabolic and reproductive dysfunction^9^. The treatment guidelines do not recommend any specific dietary intervention for PCOS^1^. Despite this, the Mediterranean diet is often suggested to patients with PCOS^10–12^, because it contains a higher proportion of fiber, unsaturated fats, and vegetables, which have anti-inflammatory properties and improve insulin sensitivity^12–15^. However, patients with PCOS have lower adherence to the Mediterranean diet compared to healthy women of a similar age and BMI^12^. One plausible explanation is that people with PCOS tend to have a shorter period of satiety, which may make it harder to follow specific diets^16^.

Existing pharmacological treatments offer temporary control of PCOS symptoms. The combined oral contraceptive pill (COCP) contains estrogen and progestogen, which is commonly prescribed for patients with PCOS who do not desire immediate fertility^17^. The COCP reduces gonadotrophins (luteinizing hormone (LH), follicle-stimulating hormone (FSH)) and androgenesis^8^. It also increases the production of SHBG, which reduces free androgens and improves menstrual regularity without inducing ovulation^18^. However, patients may avoid taking the COCP due to increased risks of thrombotic events and cervical cancer^18^. Furthermore, patients have expressed frustration with taking the COCP because it is often prescribed for extensive periods without symptomatic improvement^19^. Consequently, ineffective lifestyle advice and limited pharmacological treatments have led patients to seek alternative therapies to treat PCOS, which often lack a robust evidence base^20^.

Bioactive molecules are a potential source of treatments for PCOS. They are commonly found in foods, and their plant derivatives are called phytochemicals^21–23^, which may have the ability to alleviate illness by altering gene expression and cellular function^24^. In recent research, bioactive molecules have been shown to improve some biochemical features of PCOS, i.e., inflammation and hyperandrogenism^25^. One promising phytochemical that has been investigated for PCOS is isoflavones. Studies have shown it causes a significant reduction in oxidative stress and inflammatory markers (e.g., interleukin-6 (IL-6) and tumor necrosis factor (TNF-*α*))^26^. One study reported that a 12-week daily intervention of 50 mg of isoflavones significantly reduced insulin resistance (−0.3 ± 1.0 vs. +0.6 ± 1.1; *p* < 0.001) and free androgen index (−0.03 ± 0.04 vs. +0.02 ± 0.03; *p* < 0.001) in patients with PCOS compared to a placebo group^27^.

Women with lean PCOS may not benefit from current lifestyle recommendations that primarily emphasize weight reduction^7,17^. Dietary recommendations based on specific bioactive molecules may be more beneficial since they can focus on maximizing the micronutrients and phytonutrients that improve PCOS, whilst maintaining caloric intake. Furthermore, a bioactive molecule can often be found in several food types or in supplement form^28^, so patients can substitute foods or supplement their intake to obtain the benefits of the molecule whilst tailoring consumption to their dietary preferences.

A significant challenge to research in this field is resource constraints, which limit systematic assessment of bioactive molecules. Many potentially beneficial bioactive molecules have not progressed to clinical trials because the existing evidence for their efficacy is insufficient to drive further research^25^. Additionally, few studies to date have examined the genetic effects of bioactive molecules in PCOS, and there is a lack of understanding of the mechanisms of action of phytochemicals^25^. This hinders their progress as feasible treatment options because understanding their mechanism aids clinicians in optimizing their formulation, assessing safety, and predicting side effects.

To overcome these challenges at an exploratory stage, computational techniques such as machine learning (ML) can be used to conduct large-scale analyses of genomic data to streamline suitable candidate bioactive molecules that could alleviate symptoms of PCOS. Compared to laboratory-based or simulatory experimentation, ML may provide a quicker approach to the identification of bioactive molecules that could present therapeutic benefit in PCOS and the potential mechanisms by which these molecules exert their effects. In previous research, ML has been used to successfully identify bioactive molecules that can contribute to the management of COVID-19 infection and cancer^29–32^. Thus, we hypothesize that a similar approach could be used to identify bioactive molecules with potential therapeutic benefit for polygenic reproductive disorders, such as PCOS^5^. In this study, we use ML and graph representation learning to identify potential nutraceutical and pharmacological targets for the treatment of PCOS.

## Results

**Fig. 1:**
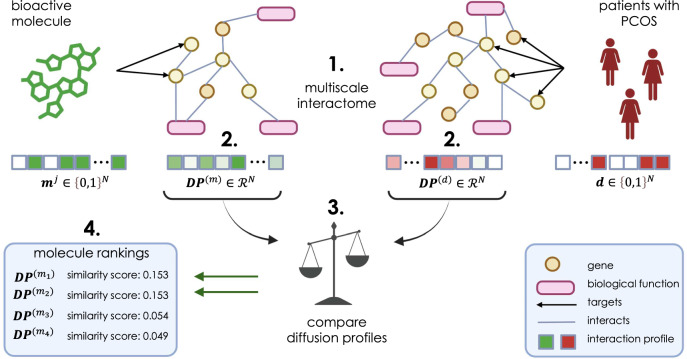
Identification of molecular targets. Assessment of the multiscale interactome and determination of whether a bioactive molecule (**m**) interacts with a disease (**d**), i.e., polycystic ovary syndrome (PCOS). The multiscale interactome (1.) contains *N* nodes (genes in orange, biological functions in pink), and edges (in gray). A biased random walk with restarts models a traversal within the network (2.), which propagates through the interactome to generate an interaction (diffusion) profile (**DP**). A diffusion profile is generated in a vectorized manner for each candidate molecule (**DP**^(*m*)^) and the target disease (**DP**^(*d*)^), PCOS. The diffusion pathways are compared (3.) using cosine similarity distance to produce a similarity score between 0 and 1; the higher the similarity score, the more likely the bioactive molecule interacts with genes related to PCOS. The bioactive molecules are ranked based on their similarity score (4.). This figure was created with BioRender.com.

In order to identify bioactive molecules that interact with the pathways responsible for the pathophysiology of PCOS, we deployed a network propagation algorithm to assess and identify bioactive molecules and pharmacological agents with high interactivity with the target disease, involving relevant genes and biological functions (Fig. 1).

### Identification of bioactive molecules

The network propagation algorithm ranked all bioactive molecules in the dataset in order of cosine similarity, which is presented in order in Supplementary Data 1^33^. This is derived based on their interactivity with thirteen genes and biological functions associated with PCOS within the multiscale interactome.

On the basis of the cosine ranking, the Elbow Detection algorithm identified a considerable drop in the cosine similarity score after the first 15 bioactive molecules. We conducted a literature search to obtain information about the top 30 bioactive molecules and/or foods in which they are present. Of these, ten bioactive molecules were identified by the algorithm that were also investigated in previous research studies (isoflavones, levoglutamide, anthocyanidin, selenomethionine, indole-3-carbinol, zinc ion, calcium, fisetin, safranal, genistein), as summarized in Table 1. Four novel bioactive molecules were identified (indole-beta-carboxylic acid, epicatechin-3-gallate, 24-methylenecycloartan-3-ol, α-Pinene), which are contained in foods that have been investigated in previous research studies involving patients with PCOS (Table 2), but the underlying bioactive molecules have not been specifically previously identified.

**Table 1:** Bioactive molecules identified by the algorithm that have been shown to present evidence of therapeutic benefit in previous studies involving patients with polycystic ovary syndrome (PCOS) or in animal models

Bioactive molecule	Algorithm ranking	Study	Subjects and intervention	Results	Level of evidence
Isoflavones	5	Jamilian et al.^27^	12-week clinical trial. Seventy people with PCOS were placed in two treatment arms: Placebo or 50 mg/day soy isoflavone.	50 mg of soy isoflavones reduced insulin levels and insulin resistance significantly (*p* < 0.001). The soy intervention group also had a lower free androgen index (*p* < 0.01).	RoB 2: Medium
		Farkhad and Khazali.^26^	32 Wistar rats were induced to have PCOS using 4 mg/kg estradiol valerate. They were split into 4 treatment groups over 21 days: control with vehicle treatment, PCOS control group with no treatment, 2 PCOS groups given soybean isoflavone-aglycone fraction (SISAF) at 50 mg/kg or 100 mg/kg.	SISAF 50 and 100 mg/g reduced IL-6, TNF-*α* and oxidative stress significantly in comparison to the PCOS control group (*p* < 0.001). The SISAF intervention decreased the number of cystic follicles in the ovaries (*p* < 0.001).	SYRCLE: Low
		Karamali et al.^36^	60 women with PCOS were either given an intervention diet (0.28 g of soy isoflavones) or a control diet over 8 weeks.	The intervention diet caused a significant reduction in plasma glucose, insulin resistance, total testosterone, and BMI (*p* < 0.02). The soy-based diet significantly raised insulin sensitivity (*p* = 0.01).	RoB 2: Low
		Li et al.^62^	51 patients with PCOS were separated into 3 intervention groups for 3 months: non-obese control group, obese control group (BMI 24 kg/m^2^), obese group with 150 mg/d puerarin for 3 months. Everyone had 1 Diane-35 tablet plus metformin 1.5 g daily.	In the puerarin treatment group, SHBG increased significantly (*p* < 0.001). Puerarin reduced testosterone and total cholesterol significantly compared to controls (*p* < 0.05).	RoB 2: Low
Levo-glutamide/ l-glutamine	6	Wu et al.^39^	40 Sprague–Dawley rats were given 6 mg dehydroepiandrosterone (DHEA) to induce PCOS. 4 rat intervention groups for 20 days: control group, PCOS induced control group, PCOS+ 0.5 g/kg Gln group or PCOS 1.0 g/kg Gln group.	0.5 g/kg glutamine caused a significant reduction in inflammatory markers: (C-reactive protein, interleukin (IL)-6, IL-18, tumor necrosis factor) compared to the control group (*p* < 0.01). In the 0.5 g/kg glutamine group, there was a significant reduction in nitric oxide (*p* < 0.001).	SYRCLE: Low
Anthocyanidin	7	Moshfegh et al.^44^	The 96 mice were induced to have PCOS with testosterone enanthate (TE). They were separated into 4 groups: 12 control (1mg/kg sesame oil), 12 TE group, 36 TE + (50,100, 600 mg/kg) SPE group, 36 TE + (20, 40, 80 mg/kg) SPA group, *n* = 12 TE removal group.	The SPE/SPA treatment caused a reduction in LH, testosterone, and estrogen. It also normalized the levels of FSH and progesterone (*p* < 0.05). Furthermore, SPA regulated the inflammatory markers (Interleukin 6, interleukin 18, TNF) and antioxidant enzymes(*p* < 0.01).	SYRCLE: Low
Selenomethionine	11	Modarres et al.^63^	40 women with fertility issues and PCOS were given 200 µg/day selenium or a placebo for 8 weeks.	Selenium supplementation of 200 µg/day for 8 weeks caused a significant reduction in fasting glucose, insulin levels and insulin resistance in patients with PCOS (*p* < 0.05).	RoB 2: Medium
		Rashidi et al.^64^	Randomized double-blinded trial, 60 participants with PCOS. Given either 8 × 109 CFU/day probiotic and 200 µg/day selenium or placebo for 12 weeks.	Probiotic and selenium co-supplementation caused a significant improvement in total antioxidant capacity. beta + 84.76 mmol/L, *p* < 0.001.	RoB 2: Medium
Indole-3-carbinol	12	Kabel et al.^65^	50 PCOS-induced Wistar rats were placed into 5 intervention groups for 36 days: Control group (no letrozole), 1 mg/kg Letrozole group, Letrozole +3 mg/kg/day oral linagliptin group, Letrozole + 50 mg/kg/day Indole 3 carbinol group, 1 mg/kg letrozole + Linagliptin.	Rats given linagliptin and/or indole-3-carbinol had a significant decrease in tissue TGF-beta1, TNF-*α*, IL-10, plasma free testosterone, luteinizing hormone, estradiol, and insulin level (*p* < 0.05).	SYRCLE: Low
Zinc ion	13	Jamilian et al.^66^	Double-blind placebo-controlled trial. For 8 weeks, 48 people with PCOS were randomly given 220 mg of zinc sulfate or a placebo drug.	The intervention group had significantly reduced alopecia and hirsutism (*p* < 0.05).	RoB 2: Medium
		Foroozanfard et al.^67^	52 patients with PCOS were separated equally into 2 two treatment arms for 8 weeks: 220 mg Zinc sulfate (containing 50 mg zinc) or a placebo group.	The intervention group had significantly reduced plasma glucose, serum insulin and insulin resistance (*p* < 0.05).	RoB 2: Medium
Calcium	14	Tehrani et al.^48^	80 people with PCOS were separated into 4 groups and given these interventions for 4 months: metformin or metformin and calcium and vitamin D, or calcium and vitamin D, or placebo.	Significant improvement in menstrual cycle regularity was noted in patients with metformin and calcium supplementation.	RoB 2: High
Fisetin	15	Mihanfar et al.^68^	24 Wistar rats were given letrozole to induce PCOS. They were separated into 4 groups: a control group (0.5% carboxymethylcellulose), PCOS group treated with letrozole (1 mg/kg), letrozole and fisetin (10 mg/kg), and letrozole and metformin (300 mg/kg).	Fisetin normalized levels of glucose, lipid profile, testosterone, estradiol, and progesterone.	SYRCLE: Low
Safranal	28	Cellat et al.^69^	32 albino Wistar rats were separated into 4 equal groups: control group given 1% carboxymethylcellulose, PCOS-induced group (given letrazole), safranal group and letrozole and Safranal group.	Safranal treatment reduced the development of cystic follicles while preserving tissue architecture.	SYRCLE: Low
Genistein	29	Khezri et al.^70^	30 Wistar rats were given either a control, 2 mg estradiol valerate (EV) to develop PCOS, 1 mg genistein or 1mg genistein and EV for PCOS.	Genistein decreased insulin resistance in EV-induced rats (*p* < 0.05). Genistein also caused normal follicular development in the ovaries, as the corpus luteum had normal histology.	SYRCLE: Low
		Romualdi et al.^71^	12 caucasian patients with obesity (27 kg/m^2^) and PCOS were given 36 mg/day genistein for 6 months.	The isoflavone intervention significantly reduced total cholesterol and LDL cholesterol level (*p* < 0.05). There were no changes to BMI, androgen levels and menstrual regularity.	RoB 2: Low

The studies were assessed for level of evidence using either the RoB 2 analytical tool for trials involving human participants or SYRCLE for trials with animals.

**Table 2:** Studies that assess candidate bioactive molecules contained in foods and their potential impact on polycystic ovary syndrome (PCOS)

Bioactive molecule			Algorithm ranking	Contained in	Study	Subjects and intervention	Results	Level of evidence
Indole-beta-carboxylic acid			1	Beetroot/common beet	Matar et al.^52^	30 women with PCOS were separated into 3 intervention groups for 3 months and were all placed on a low-carbohydrate and protein diet: 1. Control (normal medication and metformin), 2. Normal medication without metformin 3. Take 10 mg of red beetroot powder (5 mg twice daily).	The beetroot group had a significantly lower BMI compared to the control group (−4.6 kg/m^2^; *p* < 0.05).	RoB 2: Low
Epicatechin-3-gallate			3	Green tea	Tehrani et al.^48^	60 patients with PCOS with 2 intervention groups: control vs. 500 mg green tea capsules for 12 weeks.	PCOS group had significant weight loss (*p* = 0.031), as well as a reduction in free testosterone and insulin (*p* < 0.0001).	RoB 2: Medium
					Chan et al.^72^	Randomized control trial including 34 women with PCOS given either treatment with green tea capsules or a placebo for 3 months.	There was an overall 2.4% reduction in body weight in the green tea intervention group (*p* = 0.507).	RoB 2: Medium
					Farhadian et al.^73^	Double-blinded randomized control trial. 45 women with PCOS were given metformin, green tea, or a control for 3 months.	The participants given the green tea intervention had a significant reduction in weight −2.52 kg (*p* < 0.001).	RoB 2 : Medium
					Mombaini et al.^74^	Randomized control trial. 45 participants with PCOS were given green tea tablets or a placebo over 45 days.	The green tea group had a significant reduction in body weight (−1.17 kg; *p* = 0.03) and BMI (−0.47 kg/m^2^; *p* = 0.02).	RoB 2: Medium
24-Methylenecycloartan-3-ol			4	Almond	Kalgaonkar et al.^49^	31 patients with PCOS with random allocation of either 36 g of walnut or 46 g of almond intervention for 6 weeks.	Almonds resulted in a significant increase in adiponectin (*p* = 0.0262) and reduced free androgen index (*p* = 0.0470).	RoB 2: Low.
α-Pinene			10					

These studies did not attribute the therapeutic properties of these foods to their underlying molecules; however, exploratory findings from this study align with the evidence of previous studies in these instances. The studies were assessed for level of evidence using either the RoB 2 analytical tool for human participants or SYRCLE for trials involving animals.

### Categorization and validation for therapeutic benefit

Having categorized the top 30 bioactive molecules (Table 1) as being ‘not beneficial’ or of ‘potential therapeutic benefit’ in PCOS, we evaluated our algorithm for its ability to give high similarity scores for impactful bioactive molecules. Given the significant change in similarity score after the top 15 bioactive molecules, we evaluated the change in the impact of these bioactive molecules on PCOS at this cut-off. Thirteen out of 15 bioactive molecules in the top 15 (i.e., those ranked 1–15) were considered to present ‘potential therapeutic benefit’ in PCOS, while only 2 out of 15 in the next 15 candidate bioactive molecules (i.e., those ranked 16–30) were considered to present ‘potential therapeutic benefit’ in PCOS, which was statistically significant (*p* < 0.0001). These overarching categorizations are defined in the Methods, based on prior evidence.

### Localized multiscale interactome graphs of bioactive molecules

**Fig. 2:**
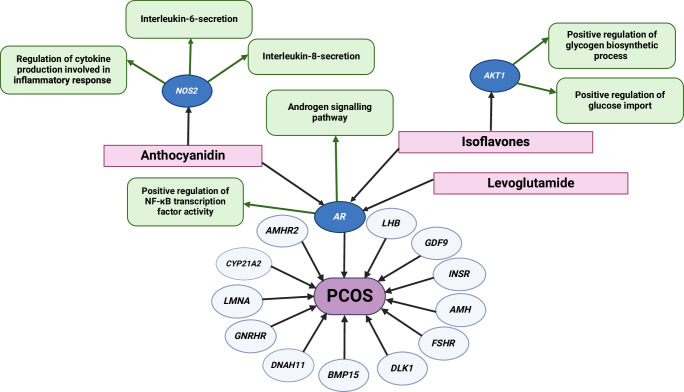
Localized multiscale interactome of known molecular targets. Localized multiscale interactome describing relevant biological functions (in green) for bioactive molecules (in pink) which were identified in the network propagation algorithm as highly influential to a subset of PCOS-related genes (*NOS2, AKT1, AR*), in dark blue ovals. Other genes known to be important in PCOS are shown in light blue ovals. These molecules have been previously shown to improve some PCOS symptoms (Table 1). This figure was created with BioRender.com.

**Fig. 3:**
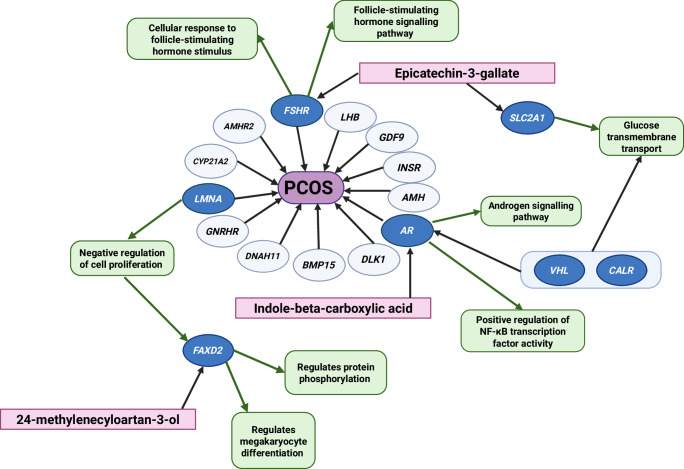
Localized multiscale interactome of novel molecular targets. Localized multiscale interactome describing biological functions (in green) for novel bioactive molecules (in pink), which were identified in the network propagation algorithm as highly influential to a subset of genes (*FSHR, LMNA, AR, FAXD2, SLC2A1, VHL, CALR*) relating to polycystic ovary syndrome (PCOS) in dark blue ovals. These molecules are contained in foods previously shown to improve some PCOS symptoms (Table 2). This figure was created with BioRender.com.

To explain why certain bioactive molecules were identified by the network propagation algorithm, we considered localized multiscale interactome (MI) graphs to seek a better understanding. Figure 2 represents a localized graph for the genes relating to PCOS, and the top three identified bioactive molecules with potential therapeutic benefit based on previous literature (anthocyanidins, isoflavones, and levoglutamide, as detailed in Table 1). These interact with genes responsible for certain biological functions, which have a potential impact on PCOS pathophysiology. This representation was also generated for novel bioactive molecules (Table 2) identified by the ML algorithm (epicatechin-3-gallate, indole-beta-carboxylic acid, and 24-methylenecycloartan-3-ol) in Fig. 3.

### Assessment of robustness to gene selection

Firstly, when considering the subset of seven genes with at least moderate evidence of association with PCOS, the network propagation algorithm performs similarly^5^. Of the top 20 bioactive molecules ranked when using thirteen genes, ten of them also appeared in the top ten when considering only the subset (Supplementary Data 1). Of the four newly identified bioactive molecules in Table 2, only epicatechin-3-gallate was identified in this sensitivity analysis, likely because *AR* was removed on the basis of evidence, and this is directly associated with the other three novel molecules. Secondly, when assessing gene overlap between genes associated with PCOS and bioactive molecules using this subset of seven genes, only seven bioactive molecules remained with non-zero similarity scores: 2-phenylethylamine, guanosine diphosphate, serotonin, pentadecanoic acid, epicatechin-3-gallate, diethylstilbesterol, and histamine. Therefore, our analysis suggests that this baseline method of gene overlap depends on polygenic diseases with many evidential gene associations to generate sufficient candidate bioactive molecules for downstream investigation.

### Predicted pharmacological agents with therapeutic benefit

In a separate analysis, we considered existing pharmacological agents (drugs) that are used in clinical practice to provide further validation of the algorithm’s potential in systematically ranking potential nutraceutical targets for PCOS. With respect to pharmacological agents that target genomic pathways involved in PCOS, the top three drug classes acted on *GNRHR*, *AR*, and *INSR*, which are recognized to be important in the pathophysiology of PCOS. When grouping pharmacological agents that are of the same class (i.e., those that target the same genes, and therefore have the same similarity score), a significant drop-off in similarity score is seen at drug ranking 77 (Supplementary Data 2). Other pharmacological agents prescribed for PCOS include metformin, which was ranked 167^th^, and progesterone, ranked 60^th^.

## Discussion

We present exploratory data using ML techniques, identifying new and established bioactive molecules that may have therapeutic interest in PCOS. Using genomic-driven ML, we identified targets that may influence genes in PCOS. We propose that this approach could be a faster and inexpensive method for the early identification of novel bioactive molecules or novel pharmaceutical candidates based on interactivity represented in the multiscale interactome^34^. Thirteen of the top fifteen ML-predicted bioactive molecules were identified as having the potential to be beneficial in PCOS, based on previously published studies. Four novel bioactive molecules identified in food items that have shown promise in improving PCOS (Table 2). As the ML algorithm is able to assess thousands of bioactive molecules efficiently, it provides quicker identification of targets with possible clinical effects, therefore streamlining the experimentation process.

In addition to the four novel bioactive molecule candidates identified, several of the other bioactive molecules identified by the ML algorithm have been previously investigated in both animal and human studies; we used this as evidence to determine the reliability of the algorithm. As significantly more bioactive molecules with potential therapeutic benefit were ranked in the top 15 molecules compared to the next 15, this suggests the algorithm bases its rankings appropriately upon the molecule’s similarity score. Thus, bioactive molecules with a higher similarity score are more likely to be of interest to relevant pathways, as they interact with more PCOS-related genes, and so have a stronger influence on the disease, as demonstrated in previous studies^29,34^. The assessment of pharmacological data provides further evidence of the reliability of the ML algorithm. Top-ranked drug classes interact with significant genes (*GNRHR, INSR, AR*) and associated pathways involved in the pathophysiology of PCOS^5^. This implies that the algorithm systematically ranks bioactive molecules or pharmacological agents based on their relative interactivity with PCOS.

The algorithm predicted that isoflavones (found in soybeans) influence PCOS through *AR*, which controls androgen receptor (AR) signaling. This ML prediction aligns with an in vitro study that demonstrated genistein (a type of isoflavone) can cause indirect AR attenuation through the estrogen receptor^35^. Therefore, isoflavones may help alleviate hyperandrogenism in PCOS. Isoflavones have also been shown to reduce insulin resistance^36^. One study reported that 30 participants with PCOS had significantly lower insulin levels when placed on a soy-based diet (−15 pmol/L; *p* < 0.001)^36^. Our localized MI for isoflavones indicates that isoflavones interact with *AKT1*, which plays an important role in insulin-stimulated glucose uptake^37^. Genistein interacts with the phosphoinositide 3-kinase/protein kinase B/mammalian target of the rapamycin pathway to increase the transcription of glucose transporter 4 (GLUT4) and facilitate glucose absorption^37,38^. Therefore, the improved insulin sensitivity observed when patients were given isoflavones may have resulted from the up-regulation of insulin-sensitive GLUT4^36^. Unlike women with obesity and PCOS, weight loss is not a suitable intervention to increase insulin sensitivity in lean women with PCOS. Therefore, isoflavones could be particularly relevant in this patient subgroup.

Our ML algorithm identified that levoglutamide (l-glutamine) interacts with *AR* to influence PCOS. l-glutamine is a non-essential amino acid that is found in barley (https://foodb.ca/), and it has been shown that 0.5 g/kg of l-glutamine significantly reduced inflammatory cytokines (IL-6, IL-18, TNF-*α*) in a DHEA-induced PCOS mouse model^39^. Interestingly, an increase in AR signaling has been shown to decrease nuclear factor-kappa b (NF-*κ*B) activity, a key stimulator of proinflammatory responses^40^. However, hyperandrogenism is a key and undesirable component of PCOS; therefore, more research is required to determine if L-glutamine has net beneficial or detrimental effects in women with PCOS.

Anthocyanidins (flavonoids found in blackcurrant) show promise in alleviating PCOS symptoms through their anti-inflammatory properties^41^. The algorithm identified that anthocyanidins interact with *NOS2*, which is known to regulate inflammatory markers through the NF-*κ*B pathway^42,43^. This ML prediction aligns with a prior animal study where saffron petal anthocyanins caused a significant decrease in inflammatory markers (TNF-*α* and IL-6) that are regulated by the NF-*κ*B pathway^44^. Although the results of this animal study may not be generalizable to women with PCOS, the ML predictions provide a potential genetic explanation for why anthocyanidins could be a therapeutic avenue for reducing inflammation in PCOS, which can be explored in future research.

Epicatechin-3-gallate is potentially the most promising newly identified phytochemical in our study, which has been identified as a predominant catechin in green tea (up to 20%)^45–47^. A meta-analysis reported green tea consumption reduced average body weight in patients with PCOS by 2.80 kg (*p* = 0.03)^47^. Another study suggested that 1 g/day of green tea caused significant weight loss and decreased testosterone in women with PCOS^48^. The ML algorithm indicated that epicatechin-3-gallate interacts with *FSHR* and *AR*, which are important reproductive hormone receptor genes and may explain some of the anti-androgenic properties of green tea^48^.

24-methylenecycloartan-3-ol is a triterpenoid present in almonds (https://foodb.ca/). In a previous study, patients with PCOS ingested 46 g of almonds daily, causing a significant increase in adiponectin, a hormone involved in regulating inflammatory markers^41,49^. Although 24-methylenecycloartan-3-ol has not been specifically investigated, other triterpenoids have been shown to possess anti-inflammatory properties similar to the effect observed with almond consumption^49,50^. Therefore, this suggests that 24-methylenecycloartan-3-ol may contribute to the anti-inflammatory effects of almonds. The ML algorithm predicted that 24-methylenecycloartan-3-ol regulates inflammation via interactions with *LMNA* and *FAXDC2*. *LMNA* produces lamin A/C, and an excess lamin A/C has been associated with a pro-inflammatory state^51^, insulin resistance, and lipodystrophy^6^. Therefore, in theory, 24-methylenecycloartan-3-ol may decrease *LMNA* activity and prevent transcription of the lamin A/C protein to exert its anti-inflammatory effect in patients with PCOS.

α-Pinene (also found in almonds) was ranked in the top fifteen bioactive molecules by the algorithm but had no direct interaction with the thirteen well-evidenced genes influencing PCOS^5^. This suggests that α-Pinene may have an indirect interaction with multiple PCOS-related genes or biological functions, giving it a higher algorithmic ranking. Therefore, the ML algorithm can predict which bioactive molecules may have an impact on PCOS through distant and/or indirect pathways, further emphasizing the potential of this ML approach, which harnesses the MI to identify novel bioactive molecules^29,34^.

Indole-beta-carboxylic acid is found in beetroot. Beetroot has been shown to significantly reduce testosterone in women with PCOS compared to controls (−7.3 pg/mL; *p* < 0.05)^52^, which aligns with our finding that indole-beta-carboxylic acid interacts with *AR*. Although no literature suggesting a direct interaction between *AR* and indole-beta-carboxylic acid was identified in our literature search, other indoles have been reported to decrease *AR* activity in prostate cancer cells^53^. Therefore, indole-beta-carboxylic acid may contribute to the anti-androgenic properties of beetroot by decreasing *AR* activity.

Our study had several limitations. Metformin is frequently used to increase insulin sensitivity in PCOS, yet it was ranked 167^th^ by the algorithm. This may be due to insufficient underlying data, as the MI suggested that metformin only targets *PRKAB1* and *ACACB*, whereas more recent research suggests the interactions of metformin are more extensive^54^. Therefore, bioactive molecules with a low ranking according to the network propagation algorithm could be due to missing gene associations with candidate molecules in the MI, since the underlying training data driven by previous experimental studies is compiled up to July 2020^34^. In the future, it would be of interest to augment the datasets to improve the predictability and quality of hypothesis generation. Another limitation is the current inability to indicate whether the interaction of candidate bioactive molecules with specific genes causes up or downregulation of the genes and whether these interactions correspond to beneficial or harmful effects. In this study, we used previous relevant studies to postulate whether the candidate bioactive molecules would have potentially therapeutic or non-beneficial effects; however, future work could include deriving a novel dataset to identify the ‘polarity’ of the interactions.

In conclusion, ML is a promising approach to identifying novel bioactive molecules that have potential therapeutic benefit in patients with PCOS, and it provides a less arduous method for predicting their mechanisms of action at a genetic level. In the future, we envisage that nutritional interventions could be a suitable adjunct to pharmacological treatment of PCOS for improved therapeutic benefit. Graph representation learning provided potential evidence for the mechanism(s) of action of three previously identified bioactive molecules (levoglutamide, anthocyanidins, and isoflavones), and four newly identified bioactive molecules (epicatechin-3-gallate, indole-beta-carboxylic acid, 24-methylenecycloartan-3-ol, α-Pinene). Further research is required to explore the therapeutic potential of these novel bioactive molecules as targets for PCOS, which now need to be tested for efficacy in clinical studies.

## Methods

### Constructing the multiscale interactome

In order to identify bioactive molecules that influence PCOS-associated genes, we used the multiscale interactome (MI)^34^. This interactome utilizes open-source datasets with information on 2100 bioactive molecules, 17,600 genes, 1508 pharmacological agents (drugs), biological functions, and diseases. The MI is presented as a heterogeneous graph *G* = (*V*, *E*) where the node set *V* encompasses protein and biological functions and the edge set *E*, represents protein–protein, protein–biological functions, and biological function–biological function interactions. Interactions with biological functions are based on Gene Ontology data, and for more details on the construction of the MI, we refer the reader to Ruiz et al.^34^. By taking into account both biological functions and proteins as well as their interactions, it was shown that drug–disease interactivity could be predicted with 40% more accuracy^34^. Additionally, the framework increased the understanding of a molecule’s mechanism of action, side effects, and efficacy in disease treatment and allows the visualization of molecule-disease interactions. Due to this success, we utilize a similar approach to search the space of food and drug molecules for potential modulators of PCOS. In a recent systematic review, Van Der Kelen et al. highlighted 16 genes associated with PCOS with varying degrees of evidence^5^, 13 of which were available in the MI: *INSR*; *AMH*; *LMNA*; AMH receptor type 2 (*AMHR2*); androgen receptor (*AR*); cytochrome P450 family 21 A2 (*CYP21A2*); FSH receptor (*FSHR*); gonadotropin-releasing hormone receptor (*GNRHR*); dynein axonemal heavy-chain 11 (*DNAH11*); bone morphogenetic protein 15 (*BMP15*); growth differentiation factor 9 (*GDF9*); LH beta (*LHB*); and delta-like non-canonical notch ligand 1 (*DLK1*). The association between PCOS and these genes allows us to represent PCOS as an *N*-dimensional vector **d** ∈ {0, 1}^*N*^ where **d**_*i*_ = 1 for these 13 highlighted genes and 0 otherwise, and *N* is the size of the interactome. Information on 2100 bioactive molecules and their targets was obtained from FoodDB and STITCH, as well as 1508 pharmacological agents so that we could also represent drugs and molecules as *N*-dimensional vectors **m** ∈ {0, 1}^*N*^ where **m**_*i*_ = 1 if protein *i* is targeted by the molecule and 0 otherwise^33,34^.

### Network propagation algorithm

It has been shown previously that just comparing the overlap of the initial proteins which are targeted between a drug *i* and disease *j* (**d**_*i*_ and **m**_*j*_) performed worse than approaches which additionally took into account interactions between the proteins and between proteins and biological functions^34^. This insight has led to a myriad of recent approaches utilizing graph representation learning to additionally incorporate information about the MI graph^29–31^. To obtain a representation of the bioactive molecules and PCOS that accounts for the graph structure, we use a network propagation algorithm based on biased random walks with restarts^34,55,56^. Initially, the network propagation starts with the nodes where **m**_*i*_ = 1 in the case of the bioactive molecules, or **d**_*i*_ = 1 for PCOS. At every step of the algorithm, the walker can restart its walk or jump to an adjacent node in the interactome. This is done with preset probabilities calculated in a previous study^34^, which are chosen to optimize the task of predicting whether a drug treats a disease. We run this algorithm for a fixed number of steps, *k* = 1000, and generate a diffusion profile, **DP** ∈ *ℜ*^*N*^, for each molecule and PCOS where **DP**_*i*_ indicates how frequently the molecule or PCOS interacts with protein *i* as it propagates on the interactome (Fig. 1). We then calculated the cosine similarity between the diffusion profiles of the bioactive molecules and that of PCOS. This gives a score in the range (0–1) and describes the degree of overlap between genes that PCOS interacts with and those that the molecule targets. A molecule with a similarity score of 1 targets all genes in the same random walk pathway as PCOS, whereas 0 indicates that the molecule does not target any gene related to PCOS (Fig. 1). We can then rank bioactive molecules based on this similarity score to highlight bioactive molecules with the potential to alleviate symptoms of PCOS.

### Validating the identified bioactive molecules

In order to validate the bioactive molecules identified by the algorithm, we first conducted a literature search to review evidence of their therapeutic benefit for PCOS. We then conducted an analysis to test whether our approach was prioritizing beneficial bioactive molecules with statistical significance. Additionally, to improve algorithmic assessment, it is important to explain the model’s output and provide a hypothesis for the mechanism of action of the predicted bioactive molecules. To achieve this, we generate localized MI graphs and use domain knowledge to visualize and attempt to explain the effects of some of the predicted bioactive molecules on PCOS. To further validate our approach, we repeated the analysis using 1500 known medications. Given that we have more information on the uses of these drugs and their mechanisms of action, we can use these as an additional control to assess the validity of the algorithm.

#### Literature review and statistical validation

Recent systematic reviews related to nutritional interventions for PCOS were assessed to generate a list of relevant bioactive molecules^25,57,58^. The ‘FooDB’ database (https://foodb.ca/) was used to find the food items containing these bioactive molecules and the relative quantities of the bioactive molecules within the food items. Electronic databases, such as ‘PubMed’, ‘ScienceDirect’, and ‘Google Scholar’, were searched, using the names of the bioactive molecules to identify preclinical and clinical studies that have evaluated their influence on PCOS. The search was limited to scientific studies in the English language. Subsequently, each study was assessed for bias by one evaluator. To remain objective, RoB 2 Cochrane’s bias and Systematic Review Centre for Laboratory Animal Experimentation (SYRCLE) critical analysis tools were used^59^. The RoB 2 tool, a revised version of the Cochrane tool, assesses for biases in randomized controlled trials with human participants^59^, and SYRCLE, based on Cochrane’s tool, assesses for biases in animal studies^60^.

Based on existing literature, the top identified ML-predicted bioactive molecules were separated into four categories: (a) “literature shows it has potential therapeutic benefit in PCOS”, (b) “yet to be independently tested, but found in food items that show potential therapeutic benefit in PCOS”, (c) “harmful in large quantities or not beneficial in PCOS”, and (d) “no literature supports its use in PCOS”. Considering this data as a contingency table (Bernoulli distribution) enables quantification of ML performance and confirms if ML is better than random at identifying beneficial bioactive molecules. The probability of success (*p*) was the bioactive molecules with overall “potential therapeutic benefit in PCOS” (categories a and b), and failure (1 − *p*) was the bioactive molecules that were “not beneficial in PCOS” (categories c and d). Fisher’s exact test was used to assess the statistical difference in the *p* and 1 − *p* of the top group and the next group of predicted bioactive molecules, with *p* < 0.05 as the predefined limit of statistical significance. The groups were identified using the Elbow Detection algorithm, which indicated a substantial drop in similarity score in the algorithmic ranking. By identifying a significant change in similarity score, the Elbow Detection algorithm provides a more interpretable and rigorous way for undertaking binary classification than previous methods, which have used arbitrary thresholding^29,30^. This is because the threshold is more sensitive to the specific distribution of ranked bioactive molecules, and therefore more appropriate when the complexity and quantity of interactions with the target disease can vary.

#### Visualizing and generating explanations

Localized MI graphs were created of the bioactive molecules predicted with ‘potential therapeutic benefit’ of those ranked in the top 15 to elucidate their potential genetic mechanism(s) of action. Our method allows us to visualize known biological pathways of PCOS and the interactions of the identified bioactive molecules. This visualization allows us to hypothesize on the mechanisms of action of these bioactive molecules, going beyond standard, uninterpretable ML prediction methods. We generated small, localized subgraphs from the MI graph where both PCOS and the predicted bioactive molecule have high interactivity. Literature search results and domain knowledge were then used to provide possible interactions that could influence PCOS using these localized networks and attempt to explain the model’s predictions.

#### Assessing robustness to gene selection

Traditionally, identifying direct overlap between genes associated with diseases and bioactive molecules is a standard baseline approach to suggest molecular candidates with potential therapeutic benefit for PCOS^34^. Since the evidence base for genes associated with PCOS varies, we compared our findings using the network propagation algorithm with this baseline approach using a subset of seven genes with at least “limited” evidence (*INSR, AMH, AMHR2, CYP21A2, DLK1, FSHR, GDF9, GNRHR*) according to a recent systematic review^5,61^. To assess the stability of the algorithm, we also compared the cosine similarity score rankings generated when using all thirteen genes as input against this subset.

#### Clinical pharmacological data

As a control, this process was repeated with clinically available drug data (pharmacological agents). This determined if the algorithm could predict first-line PCOS treatments and assessed the reliability of the ML predictions. Furthermore, the control determined if medications could potentially be repurposed to treat PCOS.

## Supplementary information

Supplementary Data

## Data Availability

All data used in the paper are publicly available. Genome data can be collected from STRING40 (https://string-db.org), UniProt (https://www.uniprot.org), COSMIC (https://cancer.sanger.ac.uk/cosmic), and NCBI Gene (https://www.ncbi.nlm.nih.gov/gene/). Drug data can be extracted from DrugBank (https://www.drugbank.ca), DrugCentral (http://drugcentral.org), and STITCH (http://stitch.embl.de). Food data can be extracted from FooDB (https://foodb.ca) and STITCH (http://stitch.embl.de). The multiscale interactome data and analysis repository from https://github.com/snap-stanford/multiscale-interactome.
